# Spatial proximity matters: A study on collaboration

**DOI:** 10.1371/journal.pone.0259965

**Published:** 2021-12-01

**Authors:** Arianna Salazar Miranda, Matthew Claudel

**Affiliations:** 1 Department of Urban Studies and Planning, Massachusetts Institute of Technology, Cambridge, MA, United States of America; 2 Department of Geography, Portland State University, Portland, Oregon, United States of America; University of Sheffield, UNITED KINGDOM

## Abstract

As scientific research becomes increasingly cross-disciplinary, many universities seek to support collaborative activity through new buildings and institutions. This study examines the impacts of spatial proximity on collaboration at MIT from 2005 to 2015. By exploiting a shift in the location of researchers due to building renovations, we evaluate how discrete changes in physical proximity affect the likelihood that researchers co-author. The findings suggest that moving researchers into the same building increases their propensity to collaborate, with the effect plateauing five years after the move. The effects are large when compared to the average rate of collaboration among pairs of researchers, which suggests that spatial proximity is an important tool to support cross-disciplinary collaborative science. Furthermore, buildings that host researchers working in the same or related fields and from multiple departments have a larger effect on their propensity to collaborate.

## 1 Introduction

A longstanding view in urban planning and management science maintains that geography and distance play a role in shaping the exchange of ideas and collaboration [1–5]. Motivated by this conviction, building and campus managers have invested vast amounts of resources in their physical spaces, transforming traditional office spaces into open floor plans with fewer walls and doors and inviting common areas with the objective to create dense and attractive environments that support interactions and chance encounters. Meanwhile, the ubiquity of communication technologies has led many to question whether proximity will continue to play a critical role. Scholarship is increasingly collaborative, but also increasingly spread across universities [6]. Some hypothesize that telecommunication technologies negate the value of physical proximity [7].

The recent COVID-19 pandemic caused a dramatic shift in workplace practices and modes of collaboration. Millions worldwide have been forced to abandon the physical office and work, study, and collaborate remotely. Some companies like Twitter have gone as far as to allow their employees to work remotely permanently [8]. The value of in-person collaboration, serendipity, and workplace environment has become a crucial question, both for knowledge organizations and cities.

Scholarship in several fields offers theoretical perspectives that explain the effect of physical proximity on collaboration, even in an era of fast and cheap long-distance communication. One prevailing view emphasizes that geographical proximity leads to collaboration because the so-called ‘tacit’ character of knowledge requires face-to-face interaction [9]. This is most often explained by the fact that tacit knowledge is hard to transmit through writing and is best-exchanged face-to-face through a range of interactions between individuals [10]. Other complementary research argues that physical proximity influences social ties because of exposure: the more proximate people are, the more likely they are to be exposed to one another, and the higher the likelihood of a new social tie between them [5, 11]. Physical proximity is particularly important for solving complex problems and promoting innovation, between new colleagues as well as prior collaborators [12–14].

This paper explores *whether physical proximity is an important determinant of collaboration within organizations*. We do so by studying over 10 years of collaborative activity among researchers in the Massachusetts Institute of Technology (MIT). The MIT campus is particularly well-fit to evaluating collaboration because the body of faculty and researchers are organized into discrete departments, labs, and research groups, which may be co-located or physically separate. Furthermore, as a technical institute, MIT prioritizes technology transfer from basic science, which requires increasingly diverse teams with varied skill sets [6, 15–20].

Researchers must confront a fundamental challenge when studying the effect of physical distance on collaboration: location is not random. In particular, researchers collaborating or seeking to collaborate might decide to locate close to one another. We address this identification challenge by exploiting a natural experiment. As a result of centralized administrative decisions about office renovations and new building openings, faculty were relocated across MIT buildings. This discrete shift allows us to estimate the causal effect of physical proximity on collaboration by looking at pairs of researchers who were moved to the same building. We exploit this variation using fixed effects models and a treatment effects framework. The treatment group includes the pairs of researchers who moved to the same building for the first time as MIT affiliates, and the control group includes pairs of MIT affiliates who have never shared the same building.

To measure collaboration, we combine research publications spanning the 2005–2015 period with MIT’s directory data. Using these data, we construct two measures of collaboration: the number of papers co-authored by a pair of researchers in each given year and a dummy for whether the pair collaborated at all in each year.

We find that moving two researchers to the same building increases their collaboration rate up to 2.7 on the third year after moving. We explain the time delay as a standard cycle of academic publication [21, 22]. The effect plateaus at 1.85 more papers per hundred pairs five years after the move. To put this in context, this means that moving researchers to a new building where they share space with 100 new colleagues increases their collaboration with these new colleagues by 0.8 papers per year. This is a large number relative to the average rate at which MIT researchers co-author papers, which is around 1 paper per year. Reassuringly, we find no evidence of pretrends in collaboration among researchers that were moved to the same building, which suggests that people who got moved to the same building were not already collaborating before the move.

One advantage of our data is that we can also explore the organizational characteristics that mediate the increase in collaboration documented above. In the second part of the paper, we explore the role of the density of researchers, the number of departments and their distribution across buildings, and the discipline affinity of researchers in a given building. We find that moving researchers into buildings that host researchers working in the same or related fields and from multiple departments can foster more collaboration.

Literature has examined the importance of (and difference between) physical proximity and organizational proximity as an influence on the likelihood of collaboration between individuals in organizations [23–25]. One explanation holds that physical proximity influences social ties because less effort is required to connect with physically closer individuals relative to individuals who are more distant [26]. Complementary evidence has shown that being proximate enhances communication [27, 28], and amplifies the quality of collaborative outcomes [29]. This finding has been replicated in various settings, including engineering offices [30, 31], and scientific offices [4]. Not only does geographical proximity play an important role in facilitating collaboration [32–34], but the inverse has also been studied: collaborators tend to be located more geographically proximate [35].

Within this literature, we most closely follow [5, 36–38]. [36] analyzes data from teachers working at five public schools and documents greater collaboration and ties among school teachers who are assigned to classrooms on the same floor. Our findings complement the work of [5], who uses similar exogenous variation to demonstrate the effect of sudden co-location on likelihood of collaboration between knowledge workers. [37] run a field experiment at the Harvard Medical School and show that researchers randomly assigned to share an information session are more likely to co-apply to grants. In line with these papers, our findings suggest a key role for proximity in facilitating collaboration within organizations. We complement these studies by emphasizing the role of physical co-location (a point we share with [36]) in fostering collaboration even among researchers in different fields. In addition, we trace collaboration patterns over time and document a persistent positive effect on the propensity to co-author (a point we share with [5]). In addition, [38] studies collaboration patterns in MIT, describing how these depend on networks, departments, and the location of researchers. Because we exploit a discrete shift in the location of researchers, our analysis brings greater explanatory power as to the causal effect of proximity on collaboration patterns.

This paper is also related to scholarship that analyzes the relationship between the physical layout and characteristics of spaces, on one hand, and interaction between individuals, on the other [39, 40]. Linear measures of distance alone miss important aspects of spatial layouts. [41] developed space syntax techniques to explicitly quantify built spaces by measuring the distance between and the experiential qualities of rooms, passageways, and public spaces. This has led to ongoing research explaining how the nuances of spatial design affect collaboration [42]. Related studies use location-tracking devices to follow individuals’ specific location within a room [40]. Architecturally sophisticated characterizations of the physical environment and detailed tracking provide rich insights, and can inform architectural design [43]. However, in this paper, we measure spatial proximity using researchers’ co-location in the same building rather than physical distance, and without great detail about the designed qualities of the space. Although it offers less nuance, our approach can be more easily replicated with large datasets from businesses or campuses.

In addition, the ‘functional’ approach to proximity proposed by [44] is particularly well suited to identify the conditions under which unexpected collaboration might happen. A study that examined an academic setting found that faculty whose offices were located along central corridors had greater co-authorship rates than did colleagues whose offices were more peripheral [39]. Other studies have explored the spatial layout designs that support collaboration in the context of the workspace. For example, recent work suggests that layout characteristics such as the percentage of floor space dedicated to shared services and amenities [45] and the visibility across different spaces [46] are associated with knowledge sharing. Although we acknowledge the value of implementing a more sophisticated characterization of the physical environment, in this paper, we measure spatial proximity using researchers’ co-location in the same building rather than physical distance. We follow this approach because offices and researchers’ allocation within buildings could be subject to particular research agendas or space allocation constraints. This is especially relevant at MIT, where office space can be designated to entire labs instead of individual researchers. For this reason, we focus on the movement of researchers to the same building rather than the precise position that researchers occupy within a given building.

Finally, this paper is related to a large body of literature documenting collaboration patterns using citations received by scientific articles and co-authorships [47, 48]. Among the strongest conclusions drawn by this line of research is the trend towards collaboration [17, 18] and an increase in scientific publication co-authorships across nearly all disciplines [19]. Science across many fields is becoming more interdisciplinary, drawing on a greater variety of skills and expertise [15, 47], and producing work with a higher impact that spans many different institutions and crosses national boundaries [49]. This paper provides evidence using scholarly output as a proxy for collaboration, to shed light on the spatial dimensions of the knowledge creation process.

The remainder of the paper is organized as follows. Section II describes the data sources and outlines how we construct the building measures for our empirical analysis. Section III introduces our empirical strategy and presents our results. Section IV is a brief discussion, and section V concludes.

## 2 Data and measurement

This section describes the data sources and main variables used in the empirical analysis. We use directory information that describes MIT affiliated faculty and combine it with data on publications from MIT affiliated faculty for the 2005-2015 period. The two databases are linked using the MIT Identification Number: a unique 9-digit numerical value assigned to each MIT affiliate, which persists through changes in affiliation over time.

The MIT Directory database includes organizational affiliation, such as school, department, or lab, as well as the location-attributes of the offices, such as building, floor, and room. To calculate the geographical proximity between MIT affiliates, we extract the office number from the MIT Directory and use it as an indicator that distinguishes if researchers share the same building or not for every year in the sample.

Because publications in peer-reviewed scientific journals are the most common form of scholarly output in a research institution, and because co-authorship is a common mode of scholarly collaboration, we use papers as a proxy for collaboration. Here, collaboration is defined as the co-authorship between any two or more affiliates during a given year. We use co-authorships as a proxy for intellectual engagement [47, 50]. The dataset includes papers published by MIT-affiliated individuals in peer-reviewed journals with DOI number identifiers, as well as the date, and authorship. This publication information is available from a comprehensive list aggregated by Academic Analytics—a non institutional affiliated data analytics company. Academic Analytics aggregates publication data from scholarly journals, for the purposes of evaluation, strategic decision-making, and benchmarking in universities. A publication may contain multiple inter-department or intra-department pairs. All department pairs are counted according to their rate of occurrence and every co-publication of two or more individuals is counted as a co-authorship. There are 878,337 MIT co-authorship instances and 38,211 papers (with unique DOIs) spanning the years 2005 to 2015. In this dataset, there are 1,417 total MIT authors; including faculty and non-faculty.

**Fig 1:**
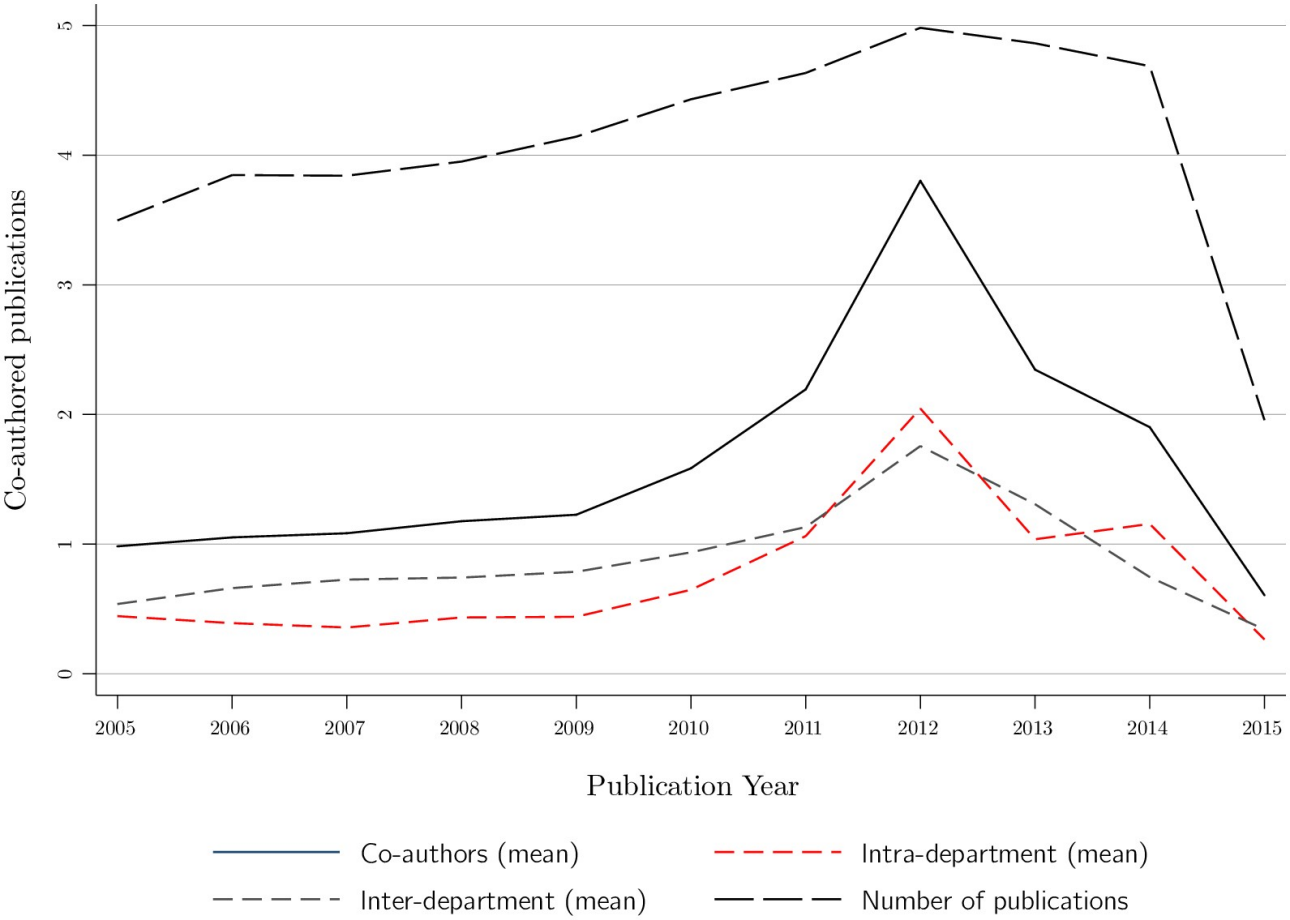
The average number of publications, co-authorships, intra-department co-authorships, and inter-department co-authorships by year.

Fig 1 plots the average number of publications among MIT affiliates over time, as well as the average number of papers that are coauthored, both with researchers within and in a different department. These figures reveal a rise in collaboration from 2005 to 2012, followed by a decline since then.

### 2.1 Measuring organizational attributes in buildings

**Fig 2:**
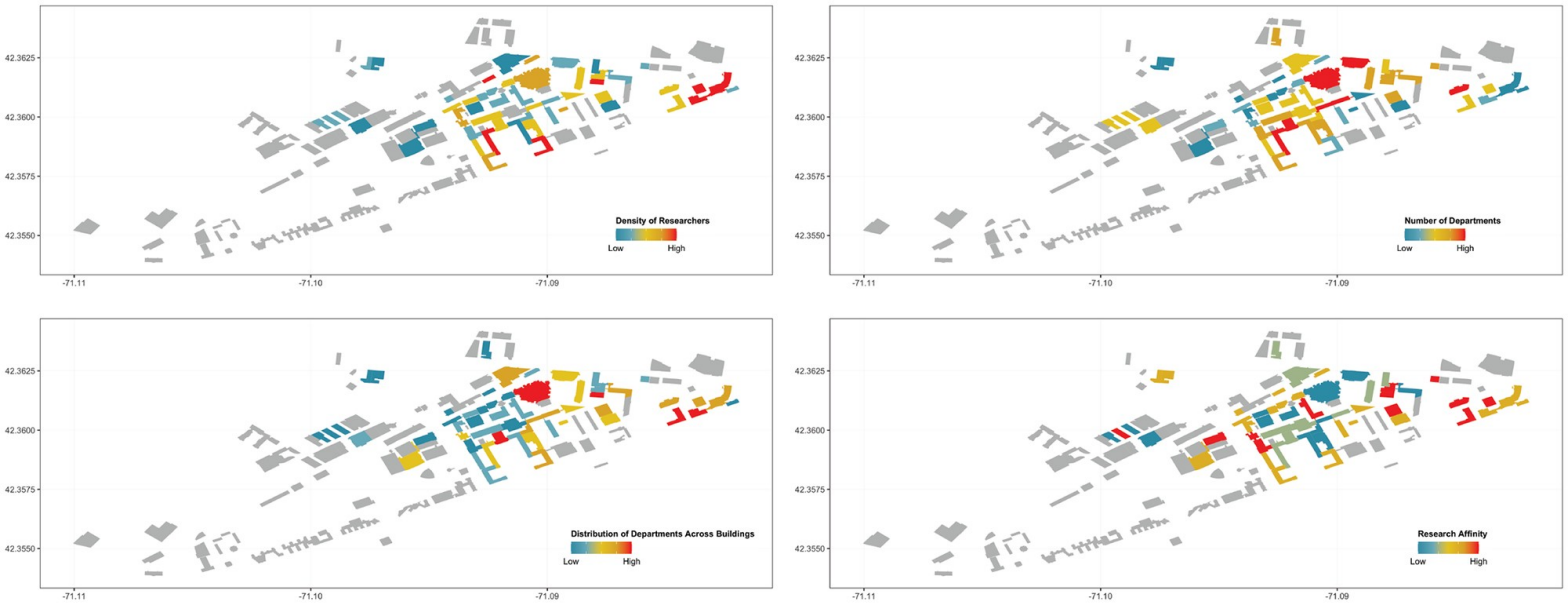
The maps show the density of researchers in each building (top left), the number of departments (top right), the distribution of departments across buildings (bottom left), and the research affinity (bottom right). Buildings shown in grey do not host researchers and are therefore not included in our sample.

The composition of researchers in buildings can contribute to the frequency and volume of communication and subsequent collaboration [4, 5, 39, 40]. Following this literature, we focus on measuring four organizational attributes that are suggested as important mediators of collaboration among researchers: the density of researchers per building, the number of departments hosted in each building and the distribution across buildings, and the discipline affinity of researchers in each building. We measure the density of researchers in each building by the number of different researchers per 100 square meters. To measure the distribution of departments across buildings we first compute for each department *d* the shares of researchers in each building, $s_{d}^{b}$, so that $\sum_{b}s_{d}^{b}=1$. We then compute the sum of these departmental shares for each building, given by $\sum_{d}s_{d}^{b}$. By construction, this measure is low when a building hosts departments that are spread across multiple buildings. Conversely, this measure is high when a building hosts departments that are concentrated in that building. To measure the discipline affinity among researchers, we match the department represented by each faculty to a set of 11 high-level disciplines. In particular, we use the network in Fig 2b proposed by [51] to define the related and unrelated disciplines. For instance, humanities are directly linked to social sciences, and social sciences are linked to mathematics and engineering. Similarly, computer science is more closely related to physics, and physics is also closely related to engineering. The disciplines included in our analysis are mathematics, computer science, physics, chemistry, engineering, earth sciences, biology, brain research, health, social science, and humanities. Since categories such as Psychology/psychiatry and Medical specialties don’t exist at MIT, we exclude them from our categorization.

Fig 2 shows the resulting four measures for each building across campus. The top panel shows the density of researchers per 100 square meters and the number of departments per building. The bottom panel shows the distribution of departments across buildings and the researchers’ discipline affinity in each building. For each measure, buildings shown in red are the highest values, and values represented in blue are the lowest.

## 3 Empirical analysis

### 3.1 Fixed effects estimator

To study the effect of proximity on collaboration, we first present fixed-effect estimates that exploit variation in the location of researchers across buildings over time. In particular, we estimate the regression model of collaboration among researcher pairs:

$$\begin{array}{c} {\text{Collaboration}}_{t,p}=\beta \cdot \text{Same}\;{\text{Building}}_{t,p}+\delta_{p}+\lambda_{t}+\varepsilon_{t,p} \end{array}$$ (1)

Here, each observation corresponds to a pair of affiliated MIT researchers, *p*, in year *t* = 2005, 2006, …, 2015, independently of whether they collaborated on that year or not. Our estimation sample includes 961 researchers and 449,055 pairs of researchers. To ensure that we are only identifying the effects of proximity by comparing existing researchers who moved to the same building, we exclude new MIT affiliates that enter the sample after 2005. The dependent variable, Collaboration_*t*,*p*_ denotes the number of papers co-authored by pair *p* during year *t*. This variable is set to zero for pairs that did not collaborate in year *t*. To facilitate the interpretation of the point estimates, we multiply the variable by 100, such that we observe papers per hundred pairs of researchers produced each year. The key explanatory variable is Same Building_*t*,*p*_, a dummy variable that indicates whether the pair of researchers are located in the same building in year *t*. *β* is the coefficient of interest, which captures the relationship between proximity and collaboration. Eq (1) assumes that collaboration by pair *p* in year *t* also depends on pair fixed effects, *δ*_*p*_, year fixed effects, *λ*_*t*_, and an error term, *ε*_*t*,*p*_.

The inclusion of pair fixed effects ensures that *β* is identified from the change in collaboration following the movement of a pair of researchers to the same building, after accounting for their baseline rate of collaboration. Treating *δ*_*p*_ as a fixed effect that must be controlled for (as opposed to a random effect) is important because one could imagine pairs with a greater propensity to collaborate sorting into the same building, which would bias our estimates of *β*.

Pair fixed effects allow for a different intercept for each pair of researchers in our sample and also control for permanent differences in collaboration across pairs. For example, pair fixed effects account for the possibility that a given pair of researchers who share a common past, have similar interests, and have compatible personalities will tend to build long-lasting collaboration relationships independently of whether they are in the same building. Pair fixed effects are also more general than a specification that explains collaboration as a function of individual researcher fixed effects. A specification with individual researcher fixed effects assumes that collaboration is given by

$$\begin{array}{c} {\text{Collaboration}}_{t,p}=\beta \cdot \text{Same}\;{\text{Building}}_{t,p}+\gamma_{i(p)}+\gamma_{j(p)}+\lambda_{t}+\varepsilon_{t,p}, \end{array}$$

where *i*(*p*) and *j*(*p*) denote the identity of the two researchers in pair *p* and *γ*_*i*(*p*)_ and *γ*_*j*(*p*)_ denote their respective fixed effects. Note that one can always define *δ*_*p*_ = *γ*_*i*(*p*)_ + *γ*_*j*(*p*)_, which implies that pair fixed effects provide a more general functional form for collaboration patterns. In particular, specifications with researcher fixed effects require their effects to be additive, ruling out complementarities or pair-specific differences in collaboration. As such, pair fixed effects account for the fact that some researchers will collaborate more with others independently of their proximity (i.e., *δ*_*p*_ is high for all pairs that include these highly collaborative individuals).

Regarding inference, we report standard errors that are two-way clustered by each researcher in a pair, *p*. This procedure recognizes that the error term *ε*_*t*,*p*_ might be correlated across pairs of researchers that have at least one researcher in common. For example, one particular researcher might have a very productive year, increasing the number of papers coauthored with some of her colleagues, and generating correlation across some of the pairs that include her. Intuitively, despite having a large number of pairs in our data, these are formed by the same 961 researchers who appear repeatedly in multiple pairs. For this reason, we cluster at the researcher level. Note that the inclusion of pair fixed effects does not ensure that the error term *ε*_*t*,*p*_ is independent across pairs nor overtime. Pair fixed effects only remove the permanent collaboration component of a pair but cannot account for other forms of correlation between pairs that have a researcher in common. For example, a positive collaboration shock might improve collaboration between A and B and also cause A to collaborate more with C in a given year, inducing correlation in the collaboration patterns of the pairs (*A*, *B*) and (*A*, *C*). This example also shows why two-way clustering at the individual researcher level is more appropriate in our context than clustering at the pair level.

Table 1 presents the estimates of Eq (1). The first panel reports the coefficients for our continuous collaboration measure (defined as papers per hundreds of pairs per year) as the dependent variable. The second panel repeats the same specifications but using a dummy variable of collaboration as the dependent variable. Here we also multiply the dependent variable by 100 to facilitate its interpretation.

**Table 1:** Estimates of the effect of proximity on collaboration.

	Dependent Variable: Collaboration					
	(1)	(2)	(3)	(4)	(5)	(6)
	Panel I. Collaboration Rate (papers per hundred pairs each year)					
Same Building	1.765***	0.786***	0.382***	0.381***	0.381***	0.374***
	(0.560)	(0.263)	(0.119)	(0.119)	(0.119)	(0.120)
Lagged Collaboration			0.348***	0.348***	0.348***	0.348***
			(0.067)	(0.067)	(0.067)	(0.067)
Implied Long-Run Effect			0.586	0.585	0.584	0.574
Observations	3357979	3325534	2874249	2874249	2874249	2874249
Number of Researchers	961	925	887	887	887	887
Number of Pairs	449055	416610	383361	383361	383361	383361
R-squared	0.00	0.49	0.61	0.61	0.61	0.61
	Panel II. Dummy for Collaboration (multiplied by 100)					
Same Building	0.707***	0.208***	0.197***	0.197***	0.196***	0.186***
	(0.114)	(0.061)	(0.064)	(0.064)	(0.064)	(0.064)
Lagged Collaboration			0.011	0.011	0.011	0.011
			(0.014)	(0.014)	(0.014)	(0.014)
Implied Long-Run Effect			0.199	0.199	0.199	0.188
Observations	3357979	3325534	2874249	2874249	2874249	2874249
Number of Researchers	961	925	887	887	887	887
Number of Pairs	449055	416610	383361	383361	383361	383361
R-squared	0.00	0.35	0.36	0.36	0.36	0.36
*Covariates*:						
Pair Fixed Effects		✓	✓	✓	✓	✓
Lagged Collaboration			✓	✓	✓	✓
Year Fixed Effects				✓	✓	✓
Department Fixed Effects					✓	✓
Same Department						✓

*Notes*: The table presents OLS estimates of the relationship between being in the same building and the collaboration rate between pairs of MIT researchers. Panel I shows results defining collaboration rates in terms of papers per hundred pairs per year. Panel II uses a dummy variable for collaboration. Column 1 presents the estimates for the baseline specification with no controls. Column 2 controls for researcher-pair fixed effects. Column 3 controls for lagged collaboration among pairs. Column 4 controls for year fixed effects. Column 5 controls for a full set of department fixed effects for both researchers in a pair. Column 6 controls for a dummy of whether the two researchers in a pair are affiliated with the same department. In parentheses, we report standard errors that are robust against heteroskedasticity and correlation within researchers across pairs.

*** denote a coefficient significant at the 1% level,

** at the 5% level, and

* at the 10% level.

Column 1 presents the estimates for the baseline specification with no controls. The estimates in column 1 show that researchers located in the same building produce 1.765 more papers per hundred of pairs each year than researchers in different buildings. Panel B shows that this is to a large extent driven by a 0.707 percentage point increase in the likelihood of collaboration among researchers in the same building relative to others. Column 2 goes one step further and controls for pair fixed effects, which ensures that our estimates are identified from the variation of researcher pairs being moved to the same building. The estimated increase in collaboration rate is now of 0.786 papers per hundred pairs. Column 3 controls for the lag value of the collaboration rate among pairs of researchers the year before they moved to the same building. This accounts for the possibility that researchers were collaborating before moving to the same building, which would bias our estimates. We find evidence of persistence in collaboration, with a coefficient of *ρ* = 0.384. The immediate effect of moving researchers to the same building is an increase in their collaboration rate of 0.382 papers per hundred pairs. Because this increase in collaboration persists, the estimates in Column 3 imply larger long-run effects of collaboration given by *β*/(1 − *ρ*) = 0.586 papers per hundred pairs, and reported at the bottom rows of the table. Finally, Columns 4, 5, and 6 control for year and building fixed effects, a full set of department fixed effects for both researchers in a pair *p*, and a dummy of whether the researchers are affiliated with the same MIT department, respectively. The inclusion of these controls does not affect our findings from Column 3.

An alternative framework for estimating Eq (1) is a random effects model. Different from a fixed effects model, in random effects, the key assumption is that the unobserved pair component *δ*_*p*_ is orthogonal to whether researchers share the same building. Random effects models are more efficient and precise but rely on this stronger assumption. Estimates of the model in column 2 via random effects deliver a point estimate of 1.133 (s.e = 0.043). This is larger than the fixed effects estimate reported in Table 1, column 2, and their difference is statistically significant at all traditional levels. This suggests that the assumptions for random effects might be violated in our context. In particular, the difference between these models suggests that pairs with a higher permanent collaboration component *δ*_*p*_ tend to sort into the same buildings underscoring the importance of controlling for pair fixed effects.

### 3.2 Treatment effects framework

To be more explicit about the control and treatment groups, we present an in-depth analysis of pairs of researchers who moved to the same building in a given year. This analysis shows how collaboration changes over time. For each year between 2006 and 2014, we define treatment and control groups as follows:

$$\begin{array}{c} T_{t,p}=\{\begin{array}{cc} 1 & \text{if}\;\text{the}\;\text{pair}\;\text{moved}\;\text{to}\;\text{the}\;\text{same}\;\text{building}\;\text{for}\;\text{the}\;\text{first}\;\text{time}\;\text{in}\;\text{year}\;t\\ & \text{and}\;\text{both}\;\text{were}\;\text{affiliated}\;\text{with}\;\text{MIT}\;\text{in}\;\text{previous}\;\text{years.}\\ 0 & \text{if}\;\text{the}\;\text{pair}\;\text{has}\;\text{never}\;\text{shared}\;\text{the}\;\text{same}\;\text{building}\;\text{but}\;\text{are}\;\text{both}\\ & \text{affiliated}\;\text{with}\;\text{MIT.} \end{array} \end{array}$$ (2)

The treatment group comprises all pairs of researchers who were not located in the same building initially but moved to the same building a given year *t*. The control group comprises all pairs of researchers who never shared the same building during the 10-year period between 2005 and 2015. Our sample excludes pairs observed in 2005 (since we do not know if this is their first year at MIT), and pairs in 2015 because we cannot trace their subsequent collaboration patterns.

Table 2 shows the number of researcher pairs by year in the treatment and control groups. The treatment group (pairs who moved to the same building in the year *t*) represents approximately 3% of the total pairs.

**Table 2:** Number of researcher pairs assigned to treatment and control groups by year.

	Number of Treated and Control Pairs by Year								
	2006	2007	2008	2009	2010	2011	2012	2013	2014
Number of Researcher	453,827	460,818	468,738	470,375	484,885	474,807	474,676	478,970	485,652
Pairs in Control Group (0)									
Number of Researcher	760	287	287	524	285	2,518	810	221	1,321
Pairs in Treated Group (1)									

*Notes*: The table presents the number of MIT researcher pairs assigned to the treatment and control groups by year. The first row summarizes the control group, which corresponds to the number of MIT researcher pairs that have never shared the same building in the 2005-2015 period. The second row summarizes the treatment group, which corresponds to the number of MIT affiliated pairs that moved to the same building for the first time in each year and were already affiliated with MIT before the move.

Using this treatment and control assignment, we estimate the following regression model:

$$\begin{array}{c} {\text{Collaboration}}_{t+h,p}=\beta_{h}\cdot T_{t,p}+\lambda_{h,t}+\varepsilon_{t,h,p}. \end{array}$$ (3)

Here, Collaboration_*t*+*h*,*p*_ denotes the number of papers co-authored by pair *p* during year *t* + *h*. We allow *h* to vary from -4 to 4 to understand how co-location relates not only to the current collaboration but also to past and future collaboration patterns. Scholarly publications are characterized by long delays to publish. The choice to focus on a 4 year time span is motivated by the fact that total average time delay from submission to publication in any field journal is 12.2 months [22]. *β*_*h*_ is the main coefficient of interest, which captures the relationship between co-location and collaboration. λ_*h*,*t*_ is a full set of year fixed effects capturing trends in collaboration over time, *t*. Finally, *ε*_*t*,*h*,*p*_ is the error term, which we again allow to be correlated within researchers across pairs.

**Fig 3:**
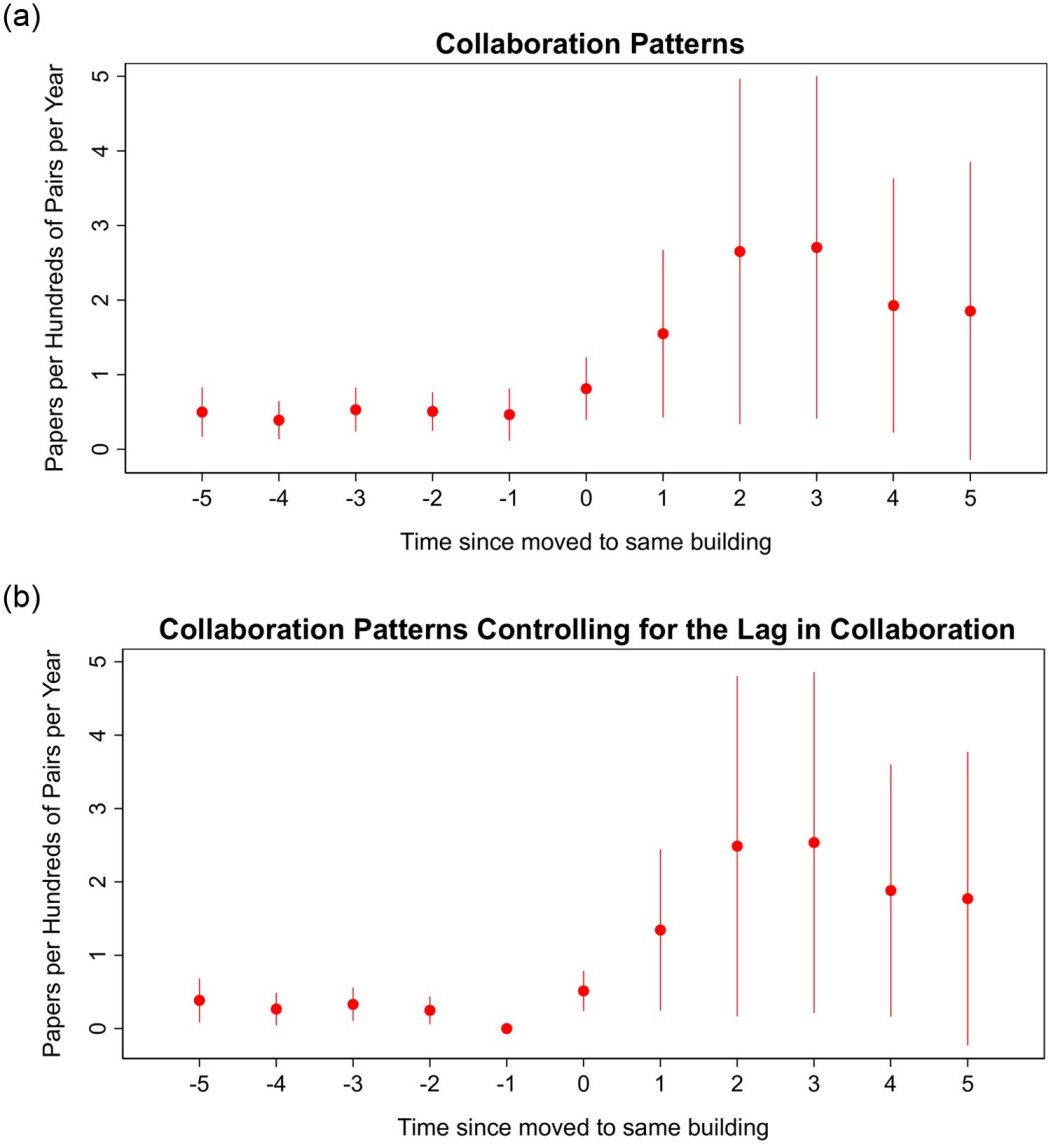
Figure showing the estimated treatment effect of moving to the same building for each year before and after the move with 90% confidence intervals. Left panel shows the estimates for *β*_*h*_ of Eq (3) and the right panel the estimates controlling for the lag of collaboration the year before treatment.

The left panel in Fig 3 plots the estimates for *β*_*h*_ for *h* = −5 to *h* = 5. Moving to the same building increases the collaboration rate between researchers by 0.8 papers per hundred pairs on the year of the move (*t* = 0). Three years after moving, the effect increases to 2.7 and plateaus at 1.85 more papers per hundred pairs five years after the move.

The right panel in Fig 3 plots the estimates for *β*_*h*_, but now controlling for lagged collaboration rates the year before the move. Moving to the same building increases the collaboration rate between researchers by 0.5 papers per hundred pairs on the first year. Three years after moving, the effect increases to 2.5 and plateaus at 1.77 more papers per hundred pairs five years after the move. The difference-in-difference results are a weighted average of all of the treatment effects estimated in Fig 3. However, the weights vary with the sample being treated each year, and hence, the magnitude of the difference-in-difference estimate is not necessarily comparable to Fig 3. This explains why the long run effect of 0.6 in the difference-in-difference exercise is lower than the 1.77 long run effect in Fig 3.

Importantly, we find no evidence of pretrends in any of these figures, suggesting that the increase in collaboration starts after the move and did not precede it.

To further bolster our identification, we now use an inverse probability score weighting to account for observed differences between pairs in the control and treatment groups. In particular, we use a logistic regression model to estimate the probability that a pair is moved to the same building as a function of the departmental affiliation of both researchers, year dummies, and their past collaboration in *t* − 1. Following [52], we then estimate the average treatment effect on the treated—ATT—by estimating Eq (3) after reweighting the data by the inverse of the propensity score. This ensures that the control group has a similar predicted probability of treatment to treated units in the reweighted sample.

**Fig 4:**
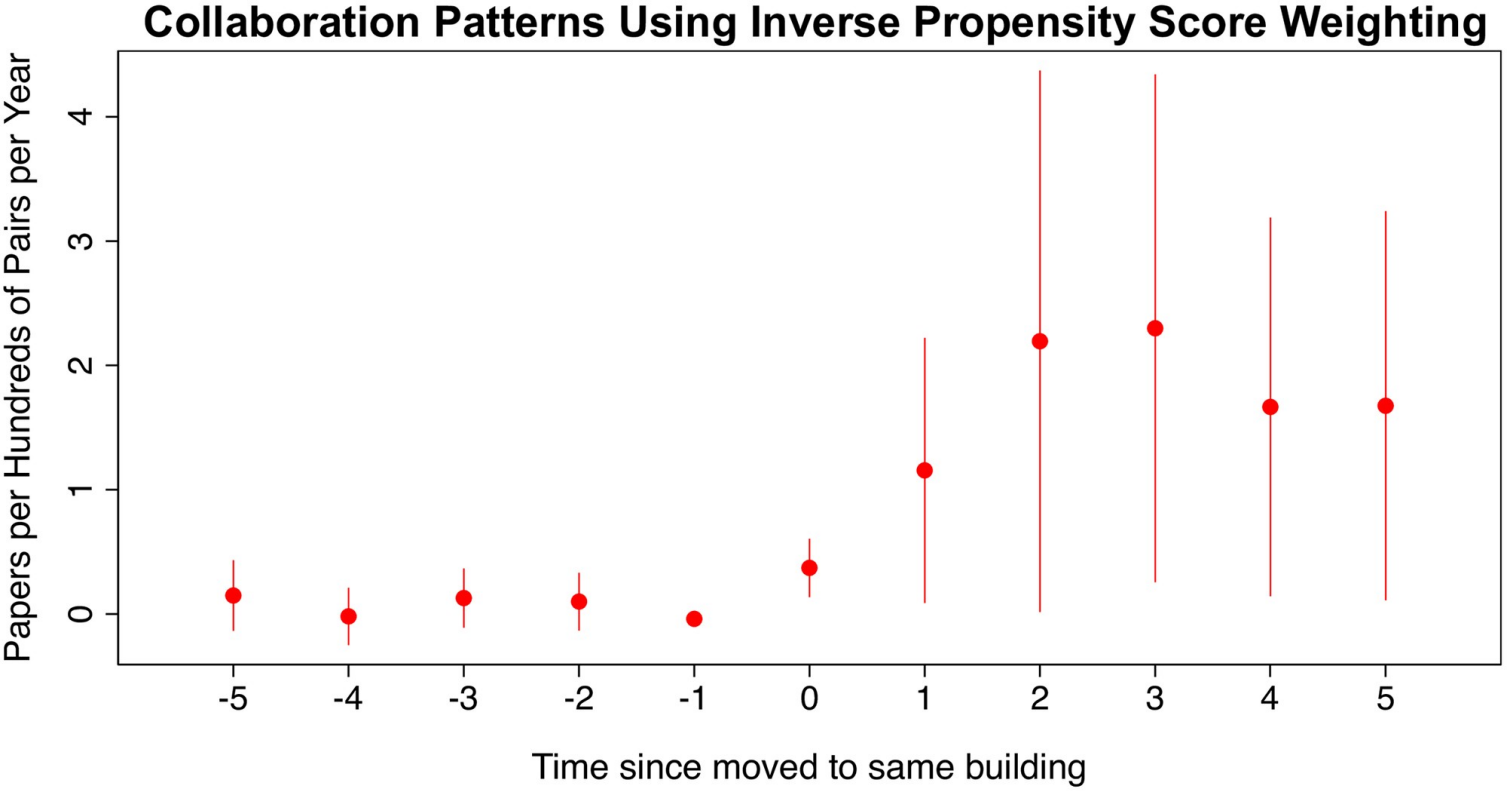
Figure showing the results from inverse propensity score reweighting. It shows the estimated treatment effect of moving to the same building for 5 years before and five years after the move (*ATT*) with 90% confidence intervals.

Fig 4 plots the estimated coefficients for the *ATT* five years before (to check for pretrends) and five years after the move. Reassuringly, the coefficients before treatment are precisely estimated zeros, which suggests that people who got moved to the same building where not already collaborating more and thus the control group is a suitable one.

Moving to the same building increases the collaboration rate between researchers by 0.37 papers per hundred pairs on the year they are moved to the same building. Three years after moving, the effect increases to 2.29 and plateaus at 1.67 more papers per hundred pairs five years after the move.

### 3.3 Building heterogeneity and collaboration

Some buildings are occupied entirely by a single department or lab, while others host a diverse group of faculty from various disciplines. In this section we explore the role of organizational arrangements in promoting collaboration.

**Fig 5:**
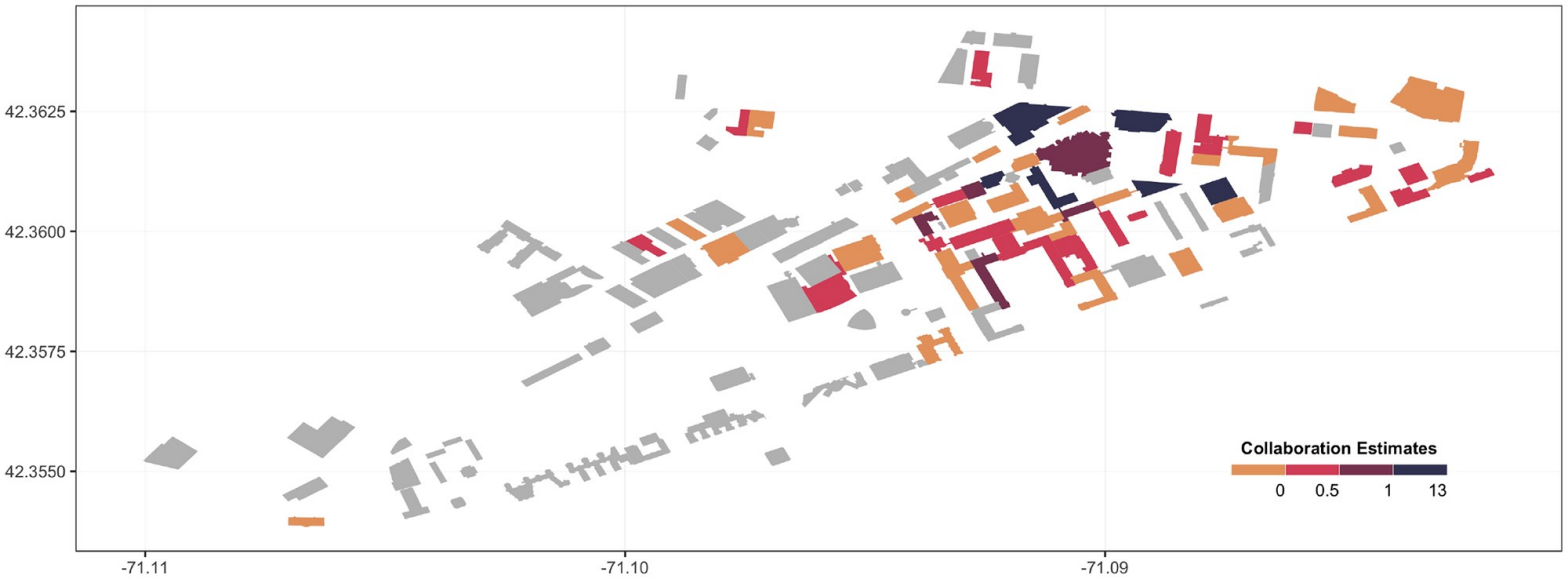
Figure showing the estimated collaboration effect for each building (Eq (1)). Buildings shown in dark purple have the highest estimated values of collaboration, and buildings shown in yellow correspond to the lowest. Buildings shown in grey do not host researchers and are therefore not included in our sample.

As a first step, we estimate Eq (1) for each building: the increase in collaboration as a result of moving two researchers to the same building. We make this estimate separately for each building on the MIT campus. Formally, this entails including a full set of interactions between the Same building_*t*,*p*_ dummy and indicators for the building hosting that pair. The point estimate on each interaction gives the gains in collaboration from moving researchers to each building. Although the building-specific estimates must be interpreted with caution due to the small sample of researchers in each building used to estimate their effect on collaboration, the estimates nevertheless suggest some heterogeneity across buildings. Fig 5 summarizes our results by plotting the building-specific estimates on collaboration using different colors. Buildings shown in dark purple have an estimated impact on collaboration above 1. Buildings in orange have an estimated impact on collaboration between 0 and 0.5. In addition, there are 21 buildings with negative but generally imprecise estimates (shown in yellow).

We now explore the differences across buildings more systematically, in order to evaluate the role of their distinct institutional arrangements. We estimate a variant of Eq (1) that allows the effect of being in the same building to vary with organizational attributes of buildings. In particular, we consider the role of four variables: the density of researchers, the number of departments and their distribution across buildings, and the affinity of fields hosted in a building. In addition, to ensure that these interactions are not confounding other differences across buildings, we control for the interaction between being in the same building and specific physical attributes of buildings, using three variables: total building area, the share of area designated for circulation, and the number of floors. These building-level attributes refer to the concept of functional zones proposed by [4].

Table 3 presents the estimates of these interactions. Column 1 explores whether the effect of being in the same building varies for pairs of researchers in the same field, related fields, or unrelated fields. Our results suggest that moving researchers to the same building produces more collaboration when they are in the same or in related fields. In particular, moving researchers to a building with others who share their same academic field increases their collaboration rate by 0.558 papers per hundred pairs relative to moving to a building with scholars working on unrelated fields (the excluded category). We also find a similar effect for researchers in related fields, although the magnitude is smaller and less precise.

**Table 3:** Interactions of the effect of proximity on organizational attributes.

	Dependent variable: Collaboration Rate			
	(1)	(2)	(3)	(4)
Same Building	0.387***	0.380***	0.382***	0.386***
	(0.123)	(0.121)	(0.119)	(0.123)
*Organizational Attributes*:				
Same Building x Same Field	0.538**	0.790**	0.860**	0.880**
	(0.248)	(0.339)	(0.347)	(0.350)
Same Building x Related Field	0.298	0.275	0.467*	0.454*
	(0.255)	(0.253)	(0.256)	(0.262)
Same Building x 4-7 Departments		0.589*	0.792**	0.730*
		(0.322)	(0.336)	(0.376)
Same Building x 7+ Departments		0.828	1.042*	0.982*
		(0.589)	(0.594)	(0.588)
Same Building x Distribution of Departments			-0.503**	-0.423
			(0.255)	(0.261)
Same Building x Researcher Density				-0.292
				(0.587)
*Building Controls*:				
Same Building x Log Total Building Area	0.216	0.080	0.122	0.091
	(0.287)	(0.242)	(0.246)	(0.267)
Same Building x Circulation Space	2.839	1.518	1.830	1.843
	(2.109)	(1.956)	(1.897)	(1.895)
Same Building x Number of Floors	-0.042	-0.029	-0.026	-0.026
	(0.064)	(0.061)	(0.063)	(0.063)
Observations	2874147	2874147	2874147	2874147
Number of Researchers	887	887	887	887
Number of Pairs	383361	383361	383361	383361
R-squared	0.61	0.61	0.61	0.61
*Covariates*:				
Pair Fixed Effects	✓	✓	✓	✓
Lagged Collaboration	✓	✓	✓	✓
Year and Building Fixed Effects	✓	✓	✓	✓

*Notes*: The table presents OLS estimates of the relationship between collaboration and the organizational attributes of buildings. In all models, we measure collaboration rates in terms of papers per hundred pairs per year. All models control for pair fixed effects, the lag of collaboration, and include year and building fixed effects. The text provides details on the construction of the organization attributes and building controls used as interactions. In parentheses, we report standard errors that are robust against heteroskedasticity and correlation within researchers across pairs.

*** denote a coefficient significant at the 1% level,

** at the 5% level, and

* at the 10% level.

Column 2 explores the role of having multiple departments within the same building. We separately estimate the effect of moving to the same building for buildings with 1–3 departments, 4–7 departments and more than 7 departments. The effects increase monotonically with the number of departments. Moving to a buildings hosting between 4 and 7 departments increases collaboration rates by 0.572 papers per hundred pairs each year relative to a building with with 1–3 departments (excluded category). Moving to a buildings hosting more than 7 departments increases collaboration rates by 0.813 papers per hundred pairs each year relative to a building with with 1–3 departments, although this effect is not precisely estimated.

Column 3 estimates the role of the distribution of departments across buildings. The estimate for this variable is negative and significant at the 10% level. The results in this column suggest that a building with multiple departments that are spread across various other buildings is more likely to foster collaboration. One potential interpretation is that researchers from departments that are spread across multiple buildings are already more open to collaborate with others.

Finally, column 4 tests for the role of the density of researchers. We estimate a negative coefficient for researcher density, but it is not statistically significant.

In sum, our results suggest that buildings that host researchers who are working in the same or related fields and from multiple departments tend to foster more collaboration. This is particularly the case for departments that are spread across multiple buildings. Other factors such as the density of researchers do not seem to play a significant role. These results should be interpreted with caution since buildings with specific organizational attributes might differ from others in terms of unobserved characteristics.

## 4 Discussion

Our findings contribute to a growing body of evidence highlighting the importance of proximity for collaboration. As a whole, there is wide agreement that proximity fosters collaboration and communication, but individual papers differ in their notion of proximity and the outcomes studied, as well as the question of whether barriers to social interaction operate across or within buildings. A first set of studies suggests that even within a building, there might be significant barriers to collaboration and social interactions. For example, [39] identifies aspects of office layouts that matter for social network formation within buildings. [36] shows that school teachers interact more when they share offices in the same floor, which points to the local nature of social interactions. Finally, [37] show that collaboration among Harvard faculty belonging to the Medicine department increases following the assignment to a shared information session, even though these faculty shared offices in the same department building. A second set of studies including [5] and our work, shows that there are gains in collaboration as a result of placing researchers in the same building. We interpret our estimates as the average effect of reducing collaboration barriers by placing researchers in the same building. It could well be the case that this average effect masks significant heterogeneity driven by differences in the layout of offices, whether researchers have offices in the same floor, the availability of common spaces (such as break rooms and cafeterias), and whether researchers use these common spaces as intended. Nonetheless, the fact that we find significant effects on collaboration just from researchers sharing the same building suggests that there are significant search costs both between and within buildings that could impede communication, collaboration, and the formation of social networks.

There are several limitations to our approach. First, as explained above, we view our estimates as an average effect, which risks missing important social interactions happening within buildings and how these are mediated by their physical design. For example, details on the presence of collaboration rooms or kitchens, and the size of staircases, could help provide a better characterization of how particular spaces within buildings mediate collaboration. A related aspect is that we measure co-location using the assigned offices in buildings. However, this definition cannot untangle between the very local effects of proximity that have been explored using concepts like functional distance and other more nuanced ways of characterizing co-location [11, 41, 43]. A second limitation is that we study the effects of collaboration for each building in isolation. This means that our findings and research design do not account for spillovers across buildings and how the reorganization of research activities across campus can affect the overall rate of collaboration at MIT. For example, some highly collaborative researchers sharing the same building might increase collaboration in that particular building but can reduce it elsewhere on campus. A better understanding of these global aspects and trade-offs is important when considering how to allocate space across departments. Finally, our research was limited to a single campus and organization, MIT.

Based on our results and identified limitations, we suggest a number of promising avenues for future research. The first is to blend our approach—using a large, long-term dataset and observing variation across relocation events—with fine-grained approaches—considering the architectural design of spaces, or conducting surveys with researchers to subjectively understand their motivations for collaborating. Subsequent work could explore a more nuanced characterization of proximity and how its effects are mediated by the physical design of buildings. Another is to do a comparative analysis of several different research institutes, or to compare different campuses of the same organization. Finally, we suggest studying the effect of full institutional closure during the COVID-19 pandemic. The effect of physical co-location on patterns of collaboration are sure to shift dramatically when all researchers are working remotely.

## 5 Conclusion

In this paper, we consider whether or not physical co-location affects the likelihood that researchers engage in scholarly collaboration. To achieve this, we exploit changes in physical proximity caused by office renovations and new building construction at the MIT campus, and take two different analytical approaches to our central question.

First, we use a treatment effects framework to explicitly define the control (pairs of researchers that never shared the same building between 2006 and 2014) and treatment groups (all pairs of researchers moved to the same building in a given year). We then estimate the treatment effect of moving to the same building on collaboration using regression methods. Second, we use inverse probability score weighting, which relies on a logistic regression model, to estimate the probability of being moved to the same building given a researchers’ department affiliation and past collaboration patterns. In particular, we find that moving researchers to the same building increase collaboration between researchers. This finding suggests that geographical co-location can help overcome barriers between departments. In the second part of the paper, we explore the role of organizational attributes of specific buildings, such as the density of researchers, the number of departments and their distribution across buildings, and the discipline affinity of researchers in a given building.

This paper provides strong empirical evidence to explain the relationship between physical co-location and the likelihood of scholarly collaboration—which is fundamental to successful scientific collaboration today. Our results suggest that buildings that host researchers who are working in the same or related fields and from multiple departments tend to foster more collaboration. We find that moving two researchers to the same building increases their collaboration rate up to 2.7 on the third year after moving. The effect plateaus at 1.85 more papers per hundred pairs five years after the move. Our results provide insights into how organizational logics for allocating space might be an important tool for building and campus planners to use as they work to design a collaborative environment—particularly relevant in the design of post-pandemic hybrid remote/on-site space use policies.
